# Nitrate ingestion blunts the increase in blood pressure during cool air exposure: a double-blind, placebo-controlled, randomized, crossover trial

**DOI:** 10.1152/japplphysiol.00593.2023

**Published:** 2024-04-04

**Authors:** Samantha N. Rowland, Emma O’Donnell, Lewis J. James, Mariasole Da Boit, Naoto Fujii, Josh T. Arnold, Alex B. Lloyd, Clare M. Eglin, Anthony I. Shepherd, Stephen J. Bailey

**Affiliations:** ^1^School of Sport, Exercise and Health Sciences, Loughborough University, Loughborough, United Kingdom; ^2^Health and Life Sciences, School of Allied Health Sciences, De Montfort University, Leicester, United Kingdom; ^3^Faculty of Health and Sport Sciences, University of Tsukuba, Ibaraki, Japan; ^4^Advanced Research Initiative for Human High Performance (ARIHHP), University of Tsukuba, Ibaraki, Japan; ^5^Norwich Medical School, Faculty of Medicine and Health Sciences, University of East Anglia, Norwich, United Kingdom; ^6^Environmental Ergonomics Research Centre, Loughborough University, Loughborough, United Kingdom; ^7^Extreme Environments Laboratory, School of Sport, Health and Exercise Science, Faculty of Science and Health, University of Portsmouth, Portsmouth, United Kingdom; ^8^Clinical Health and Rehabilitation Team, School of Sport, Health and Exercise Science, Faculty of Science and Health, University of Portsmouth, Portsmouth, United Kingdom

**Keywords:** beetroot, cardiovascular strain, inorganic nitrate, nitric oxide, thermoregulation

## Abstract

Cold exposure increases blood pressure (BP) and salivary flow rate (SFR). Increased cold-induced SFR would be hypothesized to enhance oral nitrate delivery for reduction to nitrite by oral anaerobes and to subsequently elevate plasma [nitrite] and nitric oxide bioavailability. We tested the hypothesis that dietary nitrate supplementation would increase plasma [nitrite] and lower BP to a greater extent in cool compared with normothermic conditions. Twelve males attended the laboratory on four occasions. Baseline measurements were completed at 28°C. Subsequently, participants ingested 140 mL of concentrated nitrate-rich (BR; ∼13 mmol nitrate) or nitrate-depleted (PL) beetroot juice. Measurements were repeated over 3 h at either 28°C (Norm) or 20°C (Cool). Mean skin temperature was lowered compared with baseline in PL-Cool and BR-Cool. SFR was greater in BR-Norm, PL-Cool, and BR-Cool than PL-Norm. Plasma [nitrite] at 3 h was higher in BR-Cool (592 ± 239 nM) versus BR-Norm (410 ± 195 nM). Systolic BP (SBP) at 3 h was not different between PL-Norm (117 ± 6 mmHg) and BR-Norm (113 ± 9 mmHg). SBP increased above baseline at 1, 2, and 3 h in PL-Cool but not BR-Cool. These results suggest that BR consumption is more effective at increasing plasma [nitrite] in cool compared with normothermic conditions and blunts the rise in BP following acute cool air exposure, which might have implications for attenuating the increased cardiovascular strain in the cold.

**NEW & NOTEWORTHY** Compared with normothermic conditions, acute nitrate ingestion increased plasma [nitrite], a substrate for oxygen-independent nitric oxide generation, to a greater extent during cool air exposure. Systolic blood pressure was increased during cool air exposure in the placebo condition with this cool-induced blood pressure increase attenuated after acute nitrate ingestion. These findings improve our understanding of environmental factors that influence nitrate metabolism and the efficacy of nitrate supplementation to lower blood pressure.

## Graphical abstract

**Figure:**
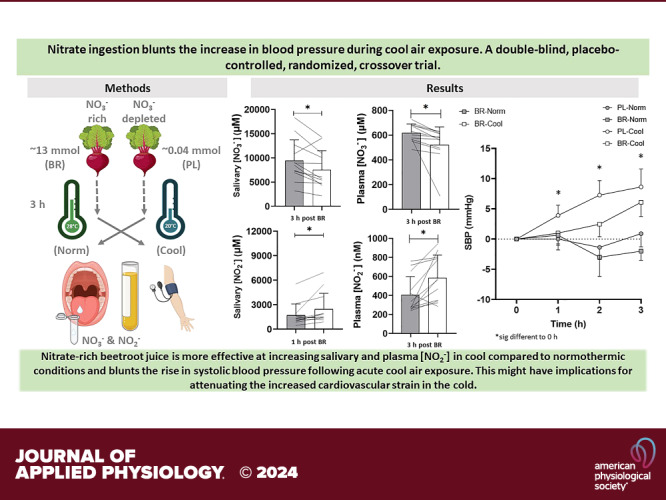

## INTRODUCTION

Hypertension is the leading cause of cardiovascular disease (CVD) and premature mortality worldwide, placing a considerable economic and healthcare burden on society (1). In response to cold stress, the sympathetic nervous system is activated and initiates peripheral vasoconstriction to minimize heat dissipation and maintain thermal balance (2, 3). Up to 40% of the local vasoconstrictive response to the cold has been attributed to lower nitric oxide production through the suppression of tonic nitric oxide/nitric oxide synthase (NOS) activity (4, 5). This cold-induced cutaneous vasoconstriction elevates cardiovascular strain through increased systemic vascular resistance and cardiac pre- and afterload, which may exacerbate hypertension and predisposition to cardiovascular events (6). Accordingly, there is marked seasonal variability in CVD mortality in high and low-latitude countries (7, 8) with an excess winter mortality rate of 17% having been observed within the UK (9).

One intervention that has been reported to lower blood pressure (BP) is dietary inorganic nitrate supplementation (10–15). The positive effects of nitrate ingestion on BP are likely mediated by its sequential reduction to nitrite and then nitric oxide via the so-called nitrate-nitrite-nitric oxide pathway (16). Briefly, following oral ingestion, nitrate is rapidly absorbed in the upper gastrointestinal tract and enters the systemic circulation (17). Approximately 25% of this exogenous nitrate is taken up by the salivary glands, via the transporter, sialin (18), and secreted in saliva (19). Subsequently, bacterial anaerobes in the mouth reduce nitrate to nitrite (20, 21). Salivary nitrite is then swallowed and further reduced to nitric oxide and other reactive nitrogen intermediates in the acidic environment of the stomach (22). A small proportion of nitrite re-enters the systemic circulation and can be reduced to nitric oxide via numerous nitrite reductases (16). This pathway, and the resultant nitric oxide production, is highly dependent on nitrate transport into the enterosalivary circulation and the host oral microbiome for nitrate reduction (21, 23–25).

Numerous acute studies have reported an inverse relationship between salivary flow rate (SFR) and ambient temperature (26–28). Since SFR influences the metabolism of nitrate by promoting nitrate secretion into the oral cavity and exposure to the oral nitrate reductases, elevated SFR may increase oral nitrate reduction to nitrite, subsequently bolstering salivary and plasma [nitrite] from a given nitrate dose. Moreover, intraoral temperature is suggested to have a close inverse relationship with pH, with recent work suggesting that the optimal composition of oral nitrate-reducing bacteria predominantly consists of alkaliogenic species (29) and that salivary and plasma nitrite are increased to a greater extent after nitrate supplementation when oral pH is elevated (30). The reduction in systolic BP (SBP) after nitrate supplementation is inversely related to plasma nitrite (11, 31) with greater reductions observed when SBP is elevated (10, 11). Elevated SBP in cool conditions may be linked to increased sympathetic nervous system activity (2, 3) and lower NOS activity (4, 5). Dietary nitrate supplementation can inhibit sympathetically mediated vasoconstriction (32) and nitrite administration can attenuate the vasoconstriction that accompanies NOS inhibition (33), which could abate increased SBP in cool conditions. Therefore, increased SFR and salivary pH during cool exposure may enhance salivary and plasma [nitrite] and the lowering in BP after nitrate supplementation in cool, compared with normothermic conditions. However, it has also been suggested that oral nitrate reduction might be attenuated at lower temperatures due to a Q_10_ effect (34), and as such, further research is required to investigate how environmental temperature influences oral nitrate metabolism, circulating plasma [nitrite] and BP after nitrate supplementation.

Although dietary nitrate supplementation has the potential to blunt arterial vasoconstriction and the subsequent rise in BP during cool exposure, augmented vasodilation may also exacerbate peripheral heat loss and declines in skin and core temperature in cool conditions. Such physiological responses would be detrimental to thermal balance and heighten cold sensation, potentially culminating in adverse health outcomes. It has been reported that nitrate supplementation can delay shivering onset during 45 min of whole body cooling without altering cutaneous vascular conductance (CVC) (35), and does not alter CVC or skin temperature during 2–30 min of local cooling (36–38). Moreover, the impact of nitrate ingestion prior to longer duration whole body cool air exposure is of relevance because whilst extreme, acute cold insults are reflective of survival situations, prolonged exposure to cool temperatures is indicative of the microclimates experienced by the elderly, heart failure patients and hypertensive individuals at home in winter. Consequently, it is important to improve understanding of whether any BP lowering afforded by nitrate supplementation in cool conditions is offset by impairments in thermal regulation to inform recommendations for nitrate supplementation in cool environmental conditions.

The purpose of the current study was to investigate the influence of lowering environmental temperature for 3 h on nitrate metabolism and BP following acute dietary nitrate supplementation. It was hypothesized that nitrate-rich beetroot juice supplementation would increase salivary and plasma [nitrite] and lower BP to a greater extent in cool compared with normothermic conditions.

## MATERIALS AND METHODS

### Participants

Twelve healthy males (mean ± SD: age: 25 ± 3 yr, stature: 1.78 ± 0.04 m, body mass: 78 ± 9 kg) volunteered to participate in this study. Females were precluded from participating because the influence of sex hormone fluctuations across the menstrual cycle on nitric oxide metabolism was unknown at the time of recruitment and experimental data collection. None of the participants were tobacco smokers or vapers. No participants were taking any medication known to interfere with stomach acid production (e.g., proton pump inhibitors) or had any preexisting medical conditions such as hypertension or diabetes. All experimental procedures were approved by the Loughborough University Research Ethics Approvals Human Participants Sub Committee. Prior to testing, participants were fully briefed before providing written, informed consent. In the 48 h prior to each subsequent visit, participants were asked to follow and replicate a number of instructions. Specifically, all trials were completed in a fed state, and participants recorded their dietary intake 24 h prior to the first experimental visit and were asked to replicate this before all subsequent visits. Participants were asked to refrain from consuming nitrate-rich foods and to avoid caffeine and alcohol ingestion 12 h and 24 h before each test, respectively. Since SFR is reduced in a state of hypohydration (39), participants were provided with 40 mL·kg^−1^ body mass^−1^ of water to consume over the 24-h period preceding each visit to ensure they arrived euhydrated (40). Participants were required to abstain from using mouthwash 48 h prior to each visit since antibacterial mouthwash markedly blunts oral reduction of nitrate to nitrite (21). All participants were instructed to adhere to their normal exercise routine for the duration of the study but were required to avoid strenuous exercise in the 24 h before each visit. Participants were instructed to wear the same clothing (shorts and t-shirt) for each visit to minimize the extraneous impact of clothing on heat transfer and all tests were performed at the same time of day (start time between 1200 and 1400) to minimize intervisit circadian variations. Experimental data collection was performed over a 12-mo period but all visits were conducted within the same season within-participant.

### Experimental Design

Using a repeated measures design, participants reported to the laboratory on five occasions. During the first visit, participants were familiarized with all the experimental procedures. During each of the four subsequent experimental visits, baseline measures of SFR, oral temperature, subjective whole body thermal sensation, skin temperature, BP, and microvascular function (CVC) were assessed, and saliva and plasma samples were obtained in an environmental chamber (Weiss-Gallenkamp, Loughborough, UK). Ambient temperature, wet bulb globe temperature (WBGT), relative humidity, and wind speed were recorded during each visit (Kestrel 4400; Nielsen-Kellerman Co., Philadelphia). At baseline, the chamber was set at 28°C (ambient temperature: 28.2 ± 0.8°C, WBGT: 27.7 ± 1.6°C, humidity: 45.7 ± 4.6%, wind speed: 0.7 ± 0.1 m/s). Subsequently, participants ingested 2 × 70 mL of concentrated nitrate-rich (BR; ∼13 mmol nitrate) or nitrate-depleted placebo (PL; ∼0.04 mmol nitrate) beetroot juice (Beet It, James White Drinks Ltd., Ipswich, UK). Over the next 3 h, participants remained in the environmental chamber with the temperature fixed at either 28°C (normothermia - ambient temperature: 28.4 ± 0.4°C, WBGT: 28.2 ± 0.4°C, humidity: 45.6 ± 2.9%, wind speed: 0.7 ± 0.1 m/s) or 20°C (cool - ambient temperature: 20.2 ± 0.1°C, WBGT: 20.9 ± 1.0°C, humidity: 44.9 ± 0.5%, wind speed: 0.7 ± 0.0 m/s). 28°C was selected as an ambient temperature within the zone of thermoneutrality. 20°C was chosen as a mild cool stimulus and intended to mimic the microclimate vulnerable individuals may be exposed to at home in winter. Pilot work within our laboratory showed that participants could tolerate this temperature for a sustained duration, and it was accompanied by elevations in BP. Salivary, temperature, BP, and microvascular function measurements were repeated each hour, with blood samples taken 3 h post supplement ingestion. In the four experimental visits, BR and PL ingestion in normothermic (BR-Norm and PL-Norm) and cool (BR-Cool and PL-Cool) conditions were administered in a placebo-controlled, randomized, and counterbalanced crossover design. PL and BR supplement administration was double-blinded (supplement bags labeled 1 and 2 by an independent investigator).

### Measurements

#### Saliva collection.

Participants rinsed their oral cavity with tap water to remove any food debris prior to sample collection. Following 2 min rest, unstimulated saliva samples were then collected via passive drool and spit into pre-weighed sterile containers every 20 s for 2 min. After a 2 min break, this process was repeated, and samples were weighed for determination of SFR, calculated by averaging SFR values over both collection periods. Subsample 1 mL aliquots were then frozen at −80°C for later analysis of salivary [nitrate] and [nitrite]. Salivary pH was measured in duplicate using a micro-FET electrode (Sentron, Leek, The Netherlands), accepted as a 5 s stable reading on the meter. A 3-point calibration of the pH probe was undertaken prior to analysis using buffers with known pH (4.01, 7.00, 10.01). Given the temperature dependency of SFR (27, 28, 41), and that salivary [nitrate] and [nitrite] are influenced by SFR (42), salivary [nitrate] and [nitrite] data were also normalized to SFR and reported as salivary nitrate and nitrite flux per min. Analytical variation (CV_A_) for SFR = 12.9% (range: 0.2–46.8%). Biological variation (CV_B_) at baseline = 16.5% (3.8–34.3%). Critical difference (CD: smallest difference required to signify true biological change) for SFR at baseline = 37.1%.

#### Oral temperature.

Oral temperature was measured using a digital thermometer (iProven, Barendrecht, The Netherlands). The thermometer was placed into the oral cavity, with readings taken with the mouth closed. Two measures were taken at each time point, with the mean value reported.

#### Thermal sensation and skin temperature.

Participants were asked to rate their subjective whole body thermal sensation using a 20-point visual scale (43). Verbal descriptors were as follows: -10: Cold impossible to bear, -8: Very cold, shivering hard, -6: Cold, light shivering, -4: Most areas of the body feel cold, -2: Some areas of the body feel cold, 0: Neutral, 2: Some areas of the body feel warm, 4: Most areas of the body feel hot, 6: Very hot, uncomfortable, 8: Extremely hot, close to limit, 10: Heat impossible to bear. Thereafter, skin temperature was measured at 15 locations (44) using a dual-force infrared monitor (Micro-Epsilon, Ortenburg, Germany). T-shirts were removed immediately prior to the recording of trunk skin temperatures. Each site was measured twice at each measurement point to obtain a mean value, and skin temperature was subsequently calculated from the unweighted mean of the fifteen body sites as per the previous protocol (45). The measurement of forearm skin temperature from the dual-force infrared monitor has also been isolated for analysis.

#### Blood pressure and microvascular function.

Participants were required to rest supine for 10 min. Thereafter, BP of the brachial artery on the left arm was measured using an automated sphygmomanometer (Omron Healthcare, Kyoto, Japan). Five measurements were taken at 2 min intervals and the mean of the five readings was used for analysis. CV_A_ for SBP = 3.2% (0.8–9.1%). CV_B_ at baseline = 3.6% (1.1–8.0%). CD at baseline = 8.9%. CV_A_ for DBP = 4.7% (1.0–12.6%). CV_B_ at baseline = 5.4% (2.8–9.3%). CD at baseline = 13.1%. Laser Doppler flowmetry (Moor Instruments, Devon, UK) was then used to assess resting cutaneous blood flow (perfusion units; PU) in a subpopulation (*n* = 5). Cutaneous vascular conductance (CVC) was calculated by dividing laser Doppler flux by the closest temporal measurement of brachial mean arterial pressure ([1/3 SBP] + [2/3 DBP]). Flux motility standard (Moor Instruments, Devon, UK) was used to calibrate the optical probe prior to each visit. Participants were required to rest supine with a cushion under their left forearm to reduce movement artifacts. The probe was placed on the ventral side of the left forearm, more than 5 cm above the wrist avoiding visible veins and tattoos. Care was taken to measure CVC at the same location for repeated measurements, but the precise location of the laser probe and thus exact local vasculature are likely not identical. The protocol consisted of resting perfusion measures for 5 min, with the average across the 5 min duration used for analysis. Flux signals (in APU) were recorded directly using MoorSOFT data capture software for subsequent off-line analysis.

### Blood Collection

Following 10 min supine rest (46), blood samples were drawn from an antecubital vein into 6 mL lithium-heparin tubes (Sarstedt, Leicester, UK) via venepuncture. Samples were collected at baseline and 3 h post supplement ingestion. Samples were centrifuged at 3000 *g* and 4°C for 10 min, within 2 min of collection. Plasma was subsequently aliquoted into Eppendorf’s and immediately frozen at -80°C for later analysis of [nitrate] and [nitrite].

### [Nitrate] and [Nitrite] Determination

All glassware, utensils, and surfaces were rinsed thoroughly with deionized water to remove residual nitrate and nitrite prior to analysis. Plasma samples were deproteinized using zinc sulfate (ZnSO_4_)/sodium hydroxide (NaOH) precipitation prior to [nitrate] determination. First, 500 μL of 0.18 N NaOH was added to 100 μL of sample followed by 5-min incubation at room temperature. Subsequently, samples were treated with 300 μL of aqueous ZnSO_4_ (5% wt/vol) and vortexed for 30 s before undergoing an additional 10-min incubation period at room temperature. Samples were then centrifuged at 21,000 *g* for 5 min and the supernatant was removed for subsequent analysis. The [nitrate] of the deproteinized plasma sample was determined by its reduction to nitric oxide in the presence of 0.8% (wt/vol) vanadium chloride (VCl_3_) in 1 M HCl via 50 μL injections into the septum of the air-tight purge vessel. The spectral emission of electronically excited nitrogen dioxide, derived from the reaction of nitric oxide with ozone, was detected by a thermoelectrically cooled, red-sensitive photomultiplier tube housed in a gas-phase chemiluminescence nitric oxide analyzer (Sievers NOA 280i, Analytix Ltd, Durham, UK). All samples were analyzed in duplicate. The [nitrate] was determined by plotting signal (mV) area against a calibration plot of sodium nitrate standards. The [nitrite] of undiluted (nondeproteinized) plasma was determined by its reduction to nitric oxide in the presence of glacial acetic acid and aqueous sodium iodide (4% wt/vol) and calibrated using sodium nitrite standards. 100 μL injections of plasma were used for [nitrite] determination. After thawing at room temperature, saliva samples were centrifuged for 10 min at 21,000 *g* rpm and the supernatant was then removed and diluted at least 100-fold with deionized water for subsequent analysis. [Nitrate] and [nitrite] were determined from 50 μL injections, using the same reagents for the respective plasma analyses.

### Statistical Analysis

Statistical analysis was performed using SPSS version 27. One-way repeated-measures ANOVAs were used to check for baseline differences across conditions (BR-Norm, PL-Norm, BR-Cool, and PL-Cool) and to assess mean values across 1–3 h. Data containing two factors [condition × time (baseline, 1 h, 2 h, and 3 h) and mean values across 1–3 h for supplement (BR and PL) × temperature (Norm and Cool)] were analyzed using two-way repeated-measures ANOVAs. Significant ANOVA effects were followed up with post hoc paired-samples *t* tests for comparisons to baseline, with the familywise error rate controlled using Holm–Bonferroni adjustment. To calculate effect sizes, partial eta squared (η_p_^2^) was used for omnibus tests and Cohen’s *d*_z_ (*t*/√ *n*) for post hoc paired-samples *t* tests. All data are displayed as means ± SD unless otherwise stated. Statistical significance was accepted at *P* < 0.05.

## RESULTS

### Thermal Sensation and Skin Temperature

All temperature indices were consistent across conditions at baseline (all *P* > 0.05; Table 1). For thermal sensation, mean skin temperature, and forearm skin temperature there were main effects for time (all *P* < 0.01, η_p_^2^ range: 0.89–0.99), condition (all *P* < 0.01, η_p_^2^ range: 0.90–0.98) and condition × time interaction effects (all *P* < 0.01, η_p_^2^ range: 0.84–0.98). There were no main effects for supplement (all *P* > 0.05, η_p_^2^ range: 0.02–0.12) or supplement × temperature interaction effects (all *P* > 0.05, η_p_^2^ range: 0.00–0.02) for any temperature variable averaged between 1 and 3 h, respectively (Table 1).

**Table 1.** Whole body thermal sensation ratings, mean skin temperature, and forearm skin temperature

	PL-Norm	BR-Norm	PL-Cool	BR-Cool
Thermal sensation				
Baseline	1 ± 1	1 ± 1	1 ± 1	1 ± 1
1 h	1 ± 1	0 ± 1	−4 ± 2*	−4 ± 2*
2 h	0 ± 1	0 ± 1	−5 ± 2*	−5 ± 1*
3 h	0 ± 1	0 ± 1	−6 ± 2*#	−5 ± 1*#
Mean skin temperature, °C				
Baseline	32.8 ± 0.6	33.1 ± 0.4	33.0 ± 0.4	33.0 ± 0.4
1 h	32.8 ± 0.4	32.9 ± 0.4*	28.4 ± 0.5*	28.6 ± 0.5*
2 h	32.7 ± 0.3	32.8 ± 0.4*	28.1 ± 0.5*	28.2 ± 0.4*
3 h	32.8 ± 0.3	32.8 ± 0.4*	28.0 ± 0.4*#	28.0 ± 0.4*#
Mean 1–3 h	32.8 ± 0.3	32.8 ± 0.4	28.2 ± 0.4#	28.3 ± 0.5#
Forearm skin temperature, °C				
Baseline	32.6 ± 0.7	32.9 ± 0.3	32.5 ± 0.7	32.7 ± 0.5
1 h	32.4 ± 0.4	32.7 ± 0.5	27.0 ± 0.9*	27.2 ± 0.7*
2 h	32.4 ± 0.5	32.6 ± 0.6	26.2 ± 1.4*	26.9 ± 0.6*
3 h	32.4 ± 0.5	32.4 ± 0.4*	26.7 ± 0.7*#	26.6 ± 0.5*#
Mean 1–3 h	32.4 ± 0.4	32.5 ± 0.5	26.6 ± 0.8#	26.9 ± 0.5#

Values are given at baseline, and at 1, 2, and 3 h as well as the mean between 1 and 3 h following ingestion of nitrate-depleted or nitrate-rich beetroot juice in normothermic (PL-Norm and BR-Norm) and cool (PL-Cool and BR-Cool) conditions. Data are presented as group means ± SD. *Lower than baseline (*P* < 0.05). #Lower than PL-Norm and BR-Norm (*P* < 0.05).

Thermal sensation was unchanged over time in PL-Norm and BR-Norm (all *P* > 0.05). Mean skin temperature was stable over time in PL-Norm (*P* > 0.05) but declined relative to baseline at 1 (*P* = 0.04), 2 (*P* = 0.03), and 3 h (*P* < 0.05) in BR-Norm. Forearm skin temperature was unchanged from baseline to 3 h in PL-Norm (*P* > 0.05) but was reduced at 3 h compared with baseline in BR-Norm (*P* < 0.01). In PL-Cool and BR-Cool, thermal sensation, mean skin temperature and forearm skin temperature were lower at 1, 2, and 3 h versus baseline (all *P* < 0.01, Table 1), but no differences were observed between PL-Norm and BR-Norm or between PL-Cool and BR-Cool at any time point (all *P* > 0.05; Table 1).

### Salivary Flow Rate and pH

There were no intercondition differences in SFR or salivary pH at baseline (*P* > 0.05). There was a main effect for condition for mean SFR between 1 and 3 h (*P* < 0.01, η_p_^2^ = 0.43). Compared with PL-Norm (592 ± 196 µL·min^−1^), mean SFR was higher in BR-Norm (697 ± 246 µL·min^−1^; *P* = 0.02, *d*_z_ = 0.54), PL-Cool (723 ± 256 µL·min^−1^; *P* = 0.02, *d*_z_ = 0.67), and BR-Cool (758 ± 261 µL·min^−1^; *P* = 0.01, *d*_z_ = 0.85). Mean SFR was not different between BR-Cool versus BR-Norm (*d*_z_ = 0.59), or PL-Cool and BR-Cool (*d*_z_ = 0.35, both *P* > 0.05). There was a main effect for supplement (*P* = 0.02, η_p_^2^ = 0.41) and temperature (*P* = 0.01, η_p_^2^ = 0.47) but no supplement × temperature interaction effect (*P* > 0.05, η_p_^2^ = 0.29) for SFR averaged between 1 and 3 h. There was no main effect for condition for mean salivary pH between 1 and 3 h in PL-Norm (7.05 ± 0.14), BR-Norm (7.16 ± 0.19), PL-Cool (7.10 ± 0.16), and BR-Cool (7.16 ± 0.21; *P* > 0.05, η_p_^2^ = 0.20). There was no main effect for supplement (*P* > 0.05, η_p_^2^ = 0.26), temperature (*P* > 0.05, η_p_^2^ = 0.09), or supplement × temperature interaction effect (*P* > 0.05, η_p_^2^ = 0.10) for mean salivary pH between 1 and 3 h.

### Oral Temperature

There were no intercondition differences in oral temperature at baseline (*P* > 0.05). There was a main effect for time (*P* < 0.01, η_p_^2^: 0.77), condition (*P* < 0.01, η_p_^2^: 0.72), and condition × time interaction effect (*P* < 0.01, η_p_^2^: 0.65). There was no main effect for supplement (*P* > 0.05, η_p_^2^: 0.04) or supplement × temperature interaction effect (*P* > 0.05, η_p_^2^: 0.00) for oral temperature averaged between 1 and 3 h. Oral temperature was unchanged over time in PL-Norm and BR-Norm (*P* > 0.05). Compared with baseline (36.1 ± 0.5°C, 36.1 ± 0.4°C), oral temperature was reduced at 1 h (35.1 ± 0.9°C, 35.3 ± 0.8°C), 2 h (34.5 ± 1.0°C, 34.5 ± 1.1°C), and 3 h (34.4 ± 0.8°C, 34.3 ± 1.0°C) in PL-Cool and BR-Cool, respectively.

### Salivary [Nitrate] and [Nitrite]

**Figure 1.**
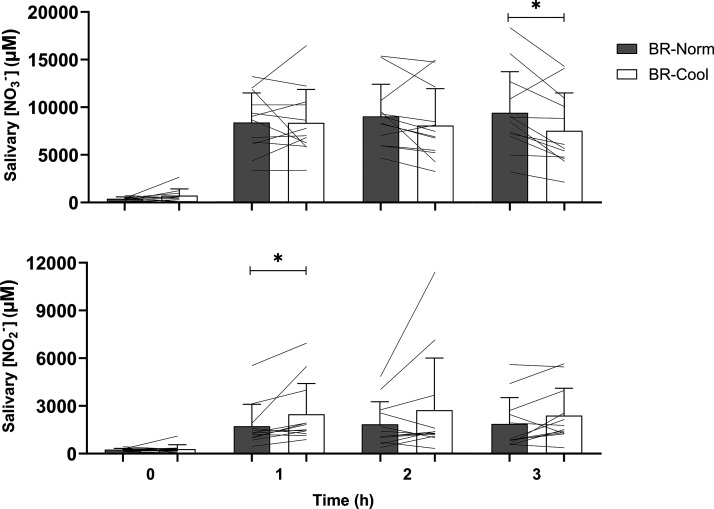
Salivary nitrate concentration ([NO_3_^−^], *upper*) and salivary nitrite concentration ([NO_2_^−^], *lower*) at baseline (0 h), 1 h, 2 h, and 3 h following ingestion of nitrate-rich beetroot juice in normothermic (BR-Norm) and cool (BR-Cool) conditions. The statistical method used was a two-way repeated-measures ANOVAs with post hoc paired-samples *t* tests with Holm–Bonferroni adjustment and are presented as group means ± SD with solid lines representing individual participants. *Difference between BR-Norm and BR-Cool (*P* < 0.05).

There were no inter-condition baseline differences in salivary [nitrate] or [nitrite], with or without normalization to SFR (all *P* > 0.05). There were main effects for time (both *P* < 0.01, η_p_^2^ = 0.80, η_p_^2^ = 0.72), condition (both *P* < 0.01, η_p_^2^ = 0.87, η_p_^2^ = 0.76), and condition × time interaction effects (both *P* < 0.01, η_p_^2^ = 0.74, η_p_^2^ = 0.67) for salivary [nitrate] and salivary [nitrate] normalized to SFR, respectively. Salivary [nitrate] was unchanged from baseline to 3 h in PL-Norm (*P* > 0.05). Absolute salivary [nitrate] was decreased relative to baseline at 1 h, and both absolute and normalized salivary [nitrate] were lower at 2 and 3 h in PL-Cool (all *P* ≤ 0.02). There were no differences between PL-Norm and PL-Cool at 1, 2, or 3 h (all *P* > 0.05). Normalizing salivary [nitrate] relative to SFR did not alter any of the observed effects in the PL conditions compared with absolute salivary [nitrate] (Table 2). There was a main effect for condition for salivary [nitrate] between 1 and 3 h (*P* < 0.01). Absolute and normalized salivary [nitrate] were higher at all time points relative to baseline in BR-Norm and BR-Cool (all *P* < 0.01), with no differences between these conditions at 1 h or 2 h (*P* > 0.05), but absolute salivary [nitrate] was higher in BR-Norm (9,459 ± 4,313 µM) versus BR-Cool (7,577 ± 3,970 µM) at 3 h (*P* = 0.04, *d*_z_ = 0.85, Fig. 1). Normalizing salivary [nitrate] to SFR removed the difference between BR-Norm and BR-Cool at 3 h (*P* > 0.05).

**Table 2.** Salivary [nitrate] and salivary [nitrite] normalized to salivary flow rate

	PL-Norm	BR-Norm	PL-Cool	BR-Cool
Salivary [NO_3_^−^], nmol·min^−1^				
Baseline	238 ± 194	241 ± 171	251 ± 142	471 ± 616
1 h	166 ± 118	5,805 ± 2,829*#	235 ± 177	6,457 ± 3,645*#
2 h	181 ± 150	6,278 ± 3,817*#	157 ± 100*	6,236 ± 4,136*#
3 h	193 ± 176	6,116 ± 3,082*#	169 ± 122*	5,442 ± 3,905*#
Mean 1–3 h	181 ± 138	6,132 ± 3,162#	186 ± 125	6,067 ± 3,832#
Salivary [NO_2_^−^], nmol·min^−1^				
Baseline	167 ± 150	153 ± 65	221 ± 192	168 ± 122
1 h	124 ± 121	1,134 ± 916*#	154 ± 139*	1,746 ± 1,170*#∼
2 h	124 ± 96	1,073 ± 663*#	188 ± 331	1,762 ± 1,624*#
3 h	135 ± 111	1,049 ± 803*#	166 ± 290	1,573 ± 1,039*#
Mean 1–3 h	127 ± 105	1,103 ± 758#	170 ± 254	1,699 ± 1,228#

Values at baseline, and at 1, 2, and 3 h for salivary nitrate concentration ([NO_3_^−^]) and salivary nitrite concentration ([NO_2_^−^]) are presented as well as the mean between 1 and 3 h following ingestion of nitrate-depleted or nitrate-rich beetroot juice in normothermic (PL-Norm and BR-Norm) and cool (PL-Cool and BR-Cool) conditions. Data are presented as group means ± SD. *Different from baseline (*P* < 0.05). ∼Higher than BR-Norm (*P* < 0.05). #Higher than PL-Norm and PL-Cool (*P* < 0.05).

There were main effects for time (both *P* < 0.01, η_p_^2^ = 0.48, η_p_^2^ = 0.59), condition (both *P* < 0.01, η_p_^2^ = 0.57, η_p_^2^ = 0.67), and condition × time interaction effects (both *P* < 0.01, η_p_^2^ = 0.42, η_p_^2^ = 0.51) for salivary [nitrite] and salivary [nitrite] normalized to SFR. There was a main effect for condition for mean salivary [nitrite] between 1 and 3 h (*P* < 0.01, η_p_^2^ = 0.57, η_p_^2^ = 0.67). Salivary [nitrite] was unchanged from baseline over 3 h in PL-Norm and PL-Cool (*P* > 0.05). Salivary [nitrite] was similar in PL-Norm and PL-Cool at 1, 2, and 3 h (all *P* > 0.05). Salivary [nitrite] was elevated above baseline between 1 and 3 h in BR-Norm and BR-Cool (all *P* ≤ 0.02), with salivary [nitrite] higher in BR-Cool versus BR-Norm at 1 h (*P* = 0.04), but no differences were observed between these conditions at 2 or 3 h (*P* > 0.05, Fig. 1). Normalizing salivary [nitrite] relative to SFR meant salivary [nitrite] was higher at 1 h versus baseline in PL-Cool (*P* = 0.03) but did not alter any of the other observed effects compared with absolute salivary [nitrite] (Table 2).

### Plasma [Nitrate] and [Nitrite]

**Figure 2.**
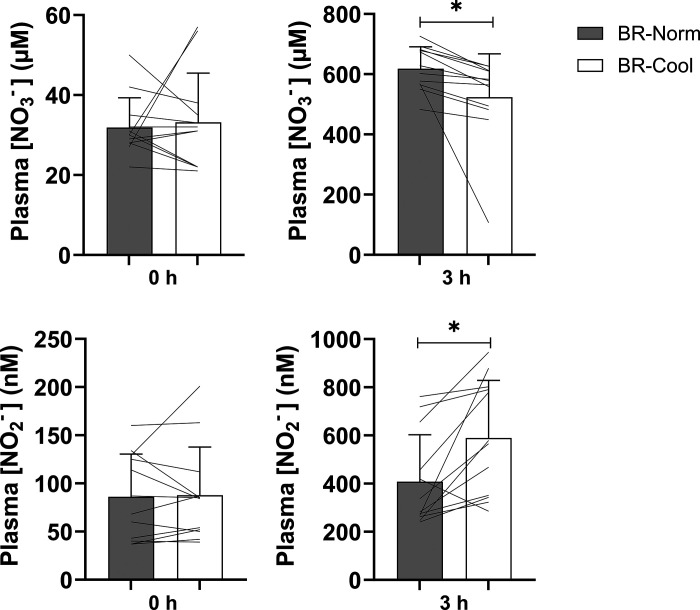
Plasma nitrate concentration ([NO_3_^−^], *upper*) and plasma nitrite concentration ([NO_2_^−^], *lower*) at baseline (0 h) and 3 h following ingestion of nitrate-rich beetroot juice in normothermic (BR-Norm) and cool (BR-Cool) conditions. The statistical method used was a two-way repeated-measures ANOVAs with post hoc paired-samples *t* tests with Holm–Bonferroni adjustment and are presented as group means ± SD with solid lines representing individual participants. *Difference between BR-Norm and BR-Cool (*P* < 0.05).

Plasma [nitrate] and [nitrite] were not different between conditions at baseline (*P* > 0.05). There was a main effect for time (*P* < 0.01, η_p_^2^ = 0.97), condition (*P* < 0.01, η_p_^2^ = 0.95), and a condition × time interaction effect (*P* < 0.01, η_p_^2^ = 0.95) for plasma [nitrate]. Plasma [nitrate] was similar in PL-Norm (25 ± 10 µM) and PL-Cool (28 ± 13 µM) at 3 h (*P* > 0.05). Plasma [nitrate] increased above baseline at 3 h in BR-Norm and BR-Cool (both *P* < 0.01), with plasma [nitrate] higher in BR-Norm (619 ± 73 µM) versus BR-Cool (524 ± 144 µM) (*P* = 0.04; *d*_z_ = 0.79, Fig. 2).

There was a main effect for time (*P* < 0.01, η_p_^2^ = 0.79), condition (*P* < 0.01, η_p_^2^ = 0.77), and a condition × time interaction effect (*P* < 0.01, η_p_^2^ = 0.77) for plasma [nitrite]. Plasma [nitrite] was similar in PL-Norm (77 ± 46 nM) and PL-Cool (85 ± 54 nM) at 3 h (*P* > 0.05) but elevated above baseline at 3 h in BR-Norm and BR-Cool (both *P* < 0.01), with plasma [nitrite] higher in BR-Cool (592 ± 239 nM) versus BR-Norm (410 ± 195 nM) (*P* = 0.01; *d*_z_ = 0.95, Fig. 2).

### Blood Pressure

**Figure 3.**
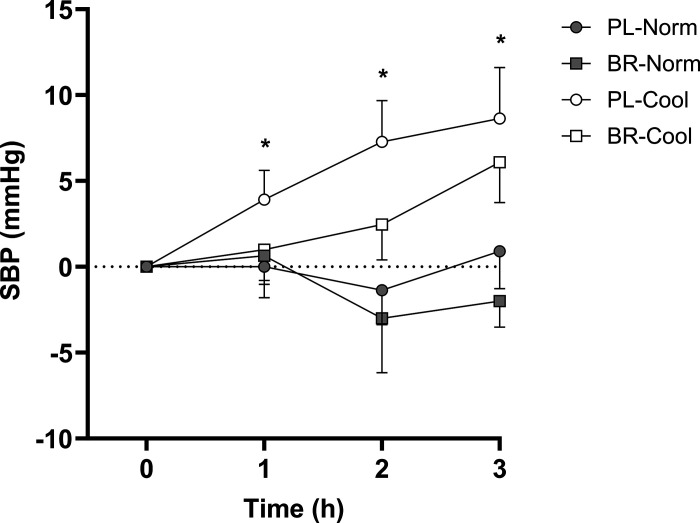
The change in brachial systolic blood pressure (SBP) from baseline (0 h), 1 h, 2 h, and 3 h post ingestion of nitrate-depleted or nitrate-rich beetroot juice in normothermic (PL-Norm, BR-Norm) and cool (PL-Cool, BR-Cool) conditions. The statistical method used was a two-way repeated-measures ANOVA with post hoc paired-samples *t* tests with Holm–Bonferroni adjustment. Data are presented as group means ± SE. *Higher than baseline in PL-Cool (*P* < 0.05), *n =* 11.

There were no differences in SBP between conditions at baseline (*P* > 0.05). There was a main effect for time (*P* = 0.01, η_p_^2^ = 0.30), condition (*P* = 0.04, η*_p_^2^* = 0.24), and a condition × time interaction effect (*P* = 0.01, η_p_^2^ = 0.22). SBP was unchanged over time in PL-Norm and BR-Norm (*P* > 0.05). SBP was elevated above baseline at 1 h (*P* < 0.05, *d*_z_ = 0.67), 2 h (*P* = 0.04, *d*_z_ = 0.88), and 3 h (*P* = 0.03, *d*_z_ = 1.05) in PL-Cool, whereas SBP was unchanged at 1 h (*d*_z_ = 0.09), 2 h (*d*_z_ = 0.24), and 3 h (*d_z_* = 0.66, all *P* > 0.05) in BR-Cool (Fig. 3). SBP at 3 h was not significantly different between BR-Norm (113 ± 9 mmHg) and PL-Norm (117 ± 6 mmHg, *d*_z_ = 0.69) or between PL-Cool (122 ± 12 mmHg) and BR-Cool (122 ± 11 mmHg; *d*_z_ = 0.08, both *P* > 0.05).

Diastolic BP (DBP) and mean arterial pressure (MAP) were not different between conditions at baseline (*P* > 0.05). There was a main effect for time (*P* < 0.01, η_p_^2^ = 0.81), condition (*P* < 0.01, η_p_^2^ = 0.77), and a condition × time interaction effect (*P* < 0.01, η_p_^2^ = 0.58) for DBP and main effects for time (*P* < 0.01, η_p_^2^ = 0.78), condition (*P* < 0.01, η_p_^2^ = 0.70) and condition × time interaction effect (*P* < 0.01, η_p_^2^ = 0.49) for MAP. DBP was unchanged over time in PL-Norm (*P* > 0.05) but increased at 3 h versus baseline in BR-Norm (*P* = 0.04). There were no differences at 3 h between PL-Norm (56 ± 6 mmHg) and BR-Norm (57 ± 6 mmHg; *P* > 0.05, *d*_z_ = 0.49, respectively). MAP was unchanged over time in PL-Norm and BR-Norm (*P* > 0.05), with no differences at 3 h between PL-Norm (76 ± 5 mmHg) and BR-Norm (76 ± 7 mmHg; *P* > 0.05, *d*_z_ = 0.09, respectively). In PL-Cool and BR-Cool, DBP and MAP were increased above baseline at 1, 2, and 3 h (all *P* < 0.01), with no differences between conditions at 3 h (70 ± 11 mmHg, 86 ± 7 mmHg vs. 70 ± 9 mmHg, 88 ± 8 mmHg; all *P* > 0.05, *d*_z_ = 0.00, *d*_z_ = 0.23, respectively).

### Microvascular Function

There were no intercondition baseline differences in skin perfusion or resting forearm CVC (*P* > 0.05). There was no main effect for time (η_p_^2^ = 0.38) or condition (η_p_^2^ = 0.23, both *P* > 0.05), but there was a condition × time interaction effect (*P* = 0.01, η_p_^2^ = 0.43) for skin perfusion. Skin perfusion was unchanged from baseline to 3 h in PL-Norm, BR-Norm, and PL-Cool (all *P* > 0.05) but reduced relative to baseline at 1 h (*P* = 0.01), 2 h (*P* = 0.02), and 3 h (*P* = 0.03) in BR-Cool (Table 3). There was a main effect of temperature for skin perfusion averaged between 1 and 3 h (*P* = 0.03, η_p_^2^ = 0.75), but post hoc analysis revealed no differences between Norm and Cool conditions. There was no main effect for supplement (*P* > 0.05, η_p_^2^ = 0.18) or supplement × temperature interaction effect (*P* > 0.05, η_p_^2^ = 0.00).

**Table 3.** Skin perfusion and forearm cutaneous vascular conductance

	PL-Norm	BR-Norm	PL-Cool	BR-Cool
Skin perfusion (flux)				
Baseline	17.26 ± 8.45	24.26 ± 9.93	22.64 ± 9.60	30.24 ± 10.36
1 h	18.72 ± 9.19	26.72 ± 7.81	11.84 ± 6.34	15.68 ± 2.44*
2 h	24.30 ± 16.26	27.20 ± 14.02	12.18 ± 7.35	15.28 ± 2.83*
3 h	22.32 ± 9.68	27.14 ± 13.08	10.24 ± 2.86	16.44 ± 2.92*
Mean 1–3 h	20.54 ± 8.26	25.35 ± 11.84	11.41 ± 4.47	15.46 ± 1.91
Skin perfusion % change from baseline				
1 h	14.8 ± 40.6	22.2 ± 55.8	−34.8 ± 47.1	−43.6 ± 19.8
2 h	39.2 ± 45.3	11.7 ± 37.1	−26.2 ± 70.6	−47.0 ± 11.5
3 h	41.6 ± 53.0	10.0 ± 25.6	−42.5 ± 38.2	−41.0 ± 23.1
CVC, flux.mmHg^−1^				
Baseline	0.23 ± 0.12	0.31 ± 0.11	0.30 ± 0.11	0.40 ± 0.12
1 h	0.25 ± 0.15	0.35 ± 0.10	0.15 ± 0.08	0.20 ± 0.04*
2 h	0.32 ± 0.21	0.34 ± 0.14	0.15 ± 0.10	0.18 ± 0.03*
3 h	0.28 ± 0.13	0.34 ± 0.14	0.12 ± 0.04∼	0.19 ± 0.04*
Mean 1–3 h	0.29 ± 0.15	0.34 ± 0.11	0.14 ± 0.05	0.19 ± 0.01
CVC % change from baseline				
1 h	17.2 ± 43.8	21.4 ± 56.6	−39.3 ± 42.2	−45.8 ± 19.9
2 h	40.3 ± 45.1	10.5 ± 37.8	−36.5 ± 61.7	−52.7 ± 12.4
3 h	41.0 ± 60.7	6.4 ± 23.9	−50.6 ± 33.4	−48.6 ± 21.0

Nitrate-depleted or nitrate-rich beetroot juice in normothermic (PL-Norm and BR-Norm) and cool (PL-Cool and BR-Cool) conditions at baseline and at 1, 2, and 3 h. Cutaneous vascular conductance (CVC; is defined as flux divided by mean arterial pressure. Mean arterial pressure was calculated as ([1/3 systolic blood pressure]/[2/3 diastolic blood pressure]). Data are presented as group means ± SD. *n* = 5 for skin perfusion and cutaneous vascular conductance. *Lower than baseline (*P* < 0.05). ∼Lower than BR-Cool (*P* < 0.05).

There was no main effect for time (*P* > 0.05, η_p_^2^ = 0.41) or condition (*P* > 0.05, η_p_^2^ = 0.39), but there was a condition × time interaction effect (*P* < 0.01, η_p_^2^ = 0.47) for CVC. CVC was unchanged from baseline to 3 h in PL-Norm, BR-Norm, and PL-Cool (all *P* > 0.05) but reduced relative to baseline at 1 h (*P* = 0.02), 2 h (*P* = 0.03), and 3 h (*P* = 0.04) in BR-Cool. There were no differences between PL-Cool and BR-Cool at 1 h (*d*_z_ = 1.03) or 2 h (*d*_z_ = 0.32), but CVC was lower in PL-Cool versus BR-Cool at 3 h (*d*_z_ = 2.75, *P* = 0.01, Table 3). There was a main effect of temperature (*P* = 0.01, η_p_^2^ = 0.83) but no main effect for supplement (*P* > 0.05, η_p_^2^ = 0.21) or supplement × temperature interaction effect (*P* > 0.05, η_p_^2^ = 0.00) for CVC averaged between 1 and 3 h. Mean CVC was not different between PL-Cool versus PL-Norm (*d*_z_ = 1.20) or BR-Cool compared with BR-Norm (*d*_z_ = 1.43, both *P* > 0.05, Table 3).

## DISCUSSION

The principal novel findings from this study were that salivary and plasma [nitrite] increased to a greater extent in BR-Cool compared with BR-Norm, and that SBP increased with time in PL-Cool, with this effect attenuated in BR-Cool. These observations are consistent with our experimental hypotheses and suggest that aspects of dietary nitrate metabolism are enhanced in cool compared with thermoneutral environments. Moreover, SBP was not reduced following BR in normothermic conditions such that BR was only effective at reducing SBP in the cool environment. Dietary nitrate supplementation may, therefore, provide a simple, low-cost intervention to lower the cardiovascular strain that accompanies cool exposure.

### Salivary Flow Rate

In line with previous studies (26–28, 47–49), SFR was increased at a lower environmental temperature in the current study. SFR was also elevated following nitrate-rich beetroot juice ingestion in normothermia. Although it has been previously suggested that nitrate-rich beetroot juice ingestion may increase SFR (50), mediated by increased nitric oxide-cyclic guanosine monophosphate signaling in salivary acinar cells (51), empirical evidence to support this is unclear (42, 52).

### Dietary Nitrate Metabolism

While salivary [nitrite] and plasma [nitrate] and [nitrite] were not different between PL-Cool and PL-Norm, salivary [nitrate] was lowered in PL-Cool compared with PL-Norm. Lower salivary [nitrate] in PL-Cool compared with PL-Norm is consistent with previous observations of lower salivary [nitrate] when SFR is increased (42). After normalizing to SFR, salivary [nitrate] was similar in PL-Cool and PL-Norm, suggesting that the cool-induced lowering in salivary [nitrate] was a function of greater SFR in cool compared with normothermic conditions.

Consistent with previous research (30, 53–55), salivary and plasma [nitrate] and [nitrite] were increased following nitrate-rich beetroot juice consumption in the current study. Plasma [nitrate] was higher 3 h post BR ingestion in BR-Norm compared with BR-Cool, whereas plasma [nitrite] was greater in BR-Cool than BR-Norm at this time point. The lower plasma [nitrate] in BR-Cool compared with BR-Norm could be linked to increased salivary nitrate uptake. Indeed, greater increases in plasma [nitrate] after BR ingestion have been reported when salivary nitrate uptake is impeded (23, 56). Increased salivary [nitrite] has been reported when SFR is elevated (57). SFR was elevated in the cool environment which may have increased salivary nitrate excretion and therefore, exposure to oral nitrate-reducing bacteria after BR ingestion. Consistent with this postulate, salivary [nitrite] and salivary [nitrite] normalized to SFR was greater after BR ingestion in BR-Cool compared with BR-Norm.

Previous research has shown that oral nitrate reduction to nitrite is greater at a higher pH (30). However, salivary pH was not augmented following cool exposure in the current study. This may suggest that the positive effects of cool temperature exposure on oral nitrate metabolism are linked to cool-induced elevations in SFR, but not changes in salivary pH. In addition to elevated salivary nitrite synthesis, plasma [nitrite] was greater in BR-Cool compared with BR-Norm such that some of the elevated salivary [nitrite] translated into higher circulating systemic [nitrite] in BR-Cool. Therefore, cool exposure appears to facilitate dietary nitrate metabolism resulting in greater increases in salivary and plasma [nitrite] post-BR ingestion when compared with normothermic conditions.

### Blood Pressure

In spite of an increase in plasma [nitrite] and enhanced potential for nitric oxide synthesis (16), SBP was not significantly lowered in BR-Norm compared with PL-Norm in the current study. This observation contrasts with some, but not all, previous work (10, 13, 58), but the magnitude of SBP lowering (−4 mmHg) 3 h post BR ingestion in BR-Norm compared with PL-Norm is consistent with previous studies reporting a significant lowering in SBP post BR ingestion in normothermic conditions (10, 13). It is possible, therefore, that the current study was statistically underpowered to detect this effect.

It is well documented that acute exposure to cool environments elevates brachial BP. Previous research studies utilizing more severe cold insults than administered in the current study have observed increases in SBP between 19 and 26 mmHg following 2-h exposure to 10°C (59) and 15 min at −15°C (60). Consistent with former studies, BP was elevated with cool air temperature exposure in the present study. In contrast to PL-Cool, where SBP increased above baseline (assessed at 28°C) after 1 h (+ 4 mmHg), 2 h (+ 7 mmHg), and 3 h (+ 9 mmHg) of rest in an environmental chamber at 20°C, SBP did not significantly increase above baseline up to 3 h in BR-Cool. Therefore, the greater increase in plasma [nitrite] and potential for nitric oxide synthesis in BR-Cool compared with BR-Norm may account for a significant offsetting of cool-induced increases in arterial BP and no effect of BR ingestion on SBP in normothermic conditions in the current study. These observations are supported by previous research suggesting that the BP reduction after nitrate supplementation is inversely related to plasma [nitrite] (11, 31) and proportionally greater when SBP is elevated (10, 11). Regarding the mechanisms for the blunted SBP increase in BR-Cool compared with PL-Cool, increased SBP during cool exposure has been attributed, at least in part, to increased sympathetic outflow (2). Increasing plasma [nitrite] can lower resting muscle sympathetic nerve activity in normotensive individuals (32) and attenuate the vasoconstriction that accompanies NOS inhibition (33). However, there is evidence to suggest that nitrate supplementation might not offset femoral artery sympathetically mediated vasoconstriction, induced by a cold-pressor test, in healthy adults (61) and it is possible that increasing plasma [nitrite] can lower BP independent of nitric oxide via an alternative redox mechanism (62). There is also evidence that the blood pressure-lowering effects might be better linked to circulating [*S*-nitrosothiols] than [nitrite] (63, 64). Given that the delivery of salivary nitrite to the stomach is an important precursor for the formation of *S*-nitrosothiols (65), it is possible that BP was lowered to a greater extent in the cool condition compared with the thermoneutral condition in the current study based on between-condition differences in salivary [nitrite] and the subsequent potential for altered circulating [*S*-nitrosothiols]. Therefore, further research is required to resolve the mechanisms for the blunted increase in BP during cool exposure after BR ingestion.

### Thermoregulatory Responses

To maintain temperature homeostasis during short-term cold exposure, the sympathetic nervous system evokes vasoconstriction and shivering thermogenesis which, respectively, decrease heat loss and increase metabolic heat production (2). In contrast, inorganic nitrate ingestion can elicit vasodilation which, if exhibited in the cutaneous microvasculature, could increase peripheral blood flow and convective and radiative heat loss, thereby compromising thermoregulation in colder environments outside the thermoneutral zone. Despite blunting the cool-induced increase in SBP, nitrate supplementation did not appear to alter CVC in the current study. This observation is consistent with previous studies reporting no effect of nitrate supplementation on cutaneous perfusion during 2–45 min cold exposure (35–38, 66), but extends these previous studies by suggesting that this may also be the case following more prolonged exposure to cool ambient temperatures. Therefore, it appears nitrate supplementation is more effective at promoting vasodilation in arteries and/or non-cutaneous microvasculature compared with the cutaneous microvasculature during whole body cooling, consistent with a recent observation that reflex cold-induced cutaneous vasoconstriction is nitric oxide independent (67). In addition, and also consistent with previous studies (35–38), forearm and mean skin temperature were not altered by nitrate supplementation in the cool environment. Thermal sensation was also not different between the PL-Cool and BR-Cool conditions in the current study. The data in the present study suggest that nitrate supplementation can offset cool-induced increases in arterial BP, thereby potentially lowering cardiac pre- and after-load. However, although there were no differences in skin temperature or thermal sensation following PL or BR ingestion in the cool condition, and in the absence of any measurements of core temperature, it is not possible to conclude that there was no clear compromise to key peripheral determinants of thermoregulation following nitrate supplementation.

### Perspectives and Significance

Our findings may have potential implications for offsetting the cardiovascular strain that accompanies cool air exposure. The cool temperature condition in the current study was designed to simulate the environment experienced in homes of high- and low-latitude countries in winter and mimicked the rise in BP that is observed during the colder months. Previous research has shown that BP is ∼5–9 mmHg higher during the winter (68, 69). Blood pressure elevations increase cardiac load and may partly explain the well-established seasonal variations in mortality and incidence of adverse health outcomes, including vascular thrombosis, arterial plaque ruptures, and arrhythmias (70). Notably, a clinical study examining seasonal variations in mortality observed that acute myocardial infarction and stroke mortality rates peak in January (relative risk ratios: 1.09 and 1.11, respectively) and are lowest in September (relative risk ratios: 0.90 and 0.91, respectively) (71). Seasonal CVD mortality may be exacerbated by a lower circulating plasma [nitrite] in the winter; in part due to reduced ultraviolet A (UVA) exposure which reduces skin NO production compared with the summer (72). Although nitrate supplementation was more effective at increasing plasma [nitrite] in the cooler condition and attenuated cool-induced increases in SBP in young normotensive adults in the current study, more research is needed to investigate whether nitrate supplementation in at-risk populations can favorably modulate cool-induced hypertension and thereby lower the incidence of cardiovascular events and mortality in the winter. This is especially important in the current unprecedented cost of living and energy crisis, which is particularly problematic for vulnerable groups in the winter.

Although skin temperature, forearm CVC, and thermal sensation were not altered after nitrate supplementation in the cool environment in the current study, it has previously been reported that nitrate supplementation delays shivering onset time and lowers the core temperature at which shivering commences in cold environments, possibly via the resetting of central thermoeffector thresholds (35). Therefore, further research is required to address the effects of nitrate supplementation on thermoregulatory responses to different degrees of cold exposure and in different populations. This is important to improve understanding of whether BP and vascular health benefits afforded by nitrate supplementation in cool conditions are offset by impairments in thermal regulation to provide a greater appreciation of the potential risk:reward ratio of nitrate supplementation in cool environments. It should be acknowledged that a limitation of the current study is that forearm skin perfusion and CVC were only assessed in a sub-population (*n* = 5) due to equipment availability and that further research is required to assess the effects of nitrate supplementation on different aspects of cardiovascular and thermal function in cool environments. Moreover, the non-forearm skin CVC responses are unknown which is important since the hands and feet are imperative for thermoregulation. Finally, BP is regulated by numerous complex mechanisms including neural, hormonal, and local factors, which were not assessed in the current study.

In conclusion, increased SBP during cool air exposure was attenuated after BR supplementation, but BR supplementation did not significantly lower SBP in normothermic conditions. BR was therefore only effective at lowering SBP in the cool condition and this was accompanied by improved dietary nitrate metabolism. Specifically, SFR was enhanced leading to greater nitrate excretion into the oral cavity and elevated salivary and plasma [nitrite] after acute BR supplementation in cool compared with normothermic conditions. These findings may have implications for attenuating the cardiovascular strain that accompanies acute cool air exposure.

## DATA AVAILABILITY

The data for this study are openly available and can be accessed at: https://doi.org/10.17028/rd.lboro.24020796.

## GRANTS

This research was supported by the National Institute for Health and Care Research (NIHR) Leicester Biomedical Research Centre.

## DISCLOSURES

No conflicts of interest, financial or otherwise, are declared by the authors.

## AUTHOR CONTRIBUTIONS

S.N.R. and S.J.B. conceived and designed research; S.N.R. and S.J.B. performed experiments; S.N.R. and S.J.B. analyzed data; S.N.R., E.O., L.J.J., M.D.B., N.F., J.T.A., A.B.L., C.M.E., A.I.S., and S.J.B. interpreted results of experiments; S.N.R. prepared figures; S.N.R. and S.J.B. drafted manuscript; S.N.R., E.O., L.J.J., M.D.B., N.F., J.T.A., A.B.L., C.M.E., A.I.S., and S.J.B. edited and revised manuscript; S.N.R., E.O., L.J.J., M.D.B., N.F., J.T.A., A.B.L., C.M.E., A.I.S., and S.J.B. approved final version of manuscript.

## References

[1] Mills KT, Stefanescu A, He J. The global epidemiology of hypertension. Nat Rev Nephrol 16: 223–237, 2020. doi:10.1038/s41581-019-0244-2. 32024986 PMC7998524

[2] Castellani JW, Young AJ. Human physiological responses to cold exposure: acute responses and acclimatization to prolonged exposure. Auton Neurosci . 196: 63–74, 2016. doi:10.1016/j.autneu.2016.02.009. 26924539

[3] Johnson JM, Minson CT, Kellogg DL Jr. Cutaneous vasodilator and vasoconstrictor mechanisms in temperature regulation. Compr Physiol 4: 33–89, 2014. doi:10.1002/cphy.c130015. 24692134

[4] Hodges GJ, Zhao K, Kosiba WA, Johnson JM. The involvement of nitric oxide in the cutaneous vasoconstrictor response to local cooling in humans. J Physiol. 574: 849–857, 2006. doi:10.1113/jphysiol.2006.109884. 16728451 PMC1817728

[5] Yamazaki F, Sone R, Zhao K, Alvarez GE, Kosiba WA, Johnson JM. Rate dependency and role of nitric oxide in the vascular response to direct cooling in human skin. J Appl Physiol (1985). 100: 42–50, 2006. doi:10.1152/japplphysiol.00139.2005. 16179403

[6] Cheng X, Su H. Effects of climatic temperature stress on cardiovascular diseases. Eur J Intern Med 21: 164–167, 2010. doi:10.1016/j.ejim.2010.03.001. 20493415

[7] Marti-Soler H, Gubelmann C, Aeschbacher S, Alves L, Bobak M, Bongard V , et al. Seasonality of cardiovascular risk factors: an analysis including over 230 000 participants in 15 countries. Heart 100: 1517–1523, 2014. doi:10.1136/heartjnl-2014-305623. 24879630

[8] Murtas R, Russo AG. Effects of pollution, low temperature and influenza syndrome on the excess mortality risk in winter 2016-2017. BMC Public Health 19: 1445, 2019. doi:10.1186/s12889-019-7788-8. 31684915 PMC6829994

[9] Ogbebor O, Odugbemi B, Maheswaran R, Patel K. Seasonal variation in mortality secondary to acute myocardial infarction in England and Wales: a secondary data analysis. BMJ Open 8: e019242, 2018. doi:10.1136/bmjopen-2017-019242. 30030309 PMC6059346

[10] Bahadoran Z, Mirmiran P, Kabir A, Azizi F, Ghasemi A. The nitrate-independent blood pressure-lowering effect of beetroot juice: a systematic review and meta-analysis. Adv Nutr 8: 830–838, 2017 [Erratum in Adv Nutr 9: 274, 2018]. doi:10.3945/an.117.016717. 29141968 PMC5683004

[11] Kapil V, Milsom AB, Okorie M, Maleki-Toyserkani S, Akram F, Rehman F, Arghandawi S, Pearl V, Benjamin N, Loukogeorgakis S, MacAllister R, Hobbs AJ, Webb AJ, Ahluwalia A. Inorganic nitrate supplementation lowers blood pressure in humans: role for nitrite-derived NO. Hypertension 56: 274–281, 2010 [Erratum in Hypertension 56: e37–e39, 2010]. doi:10.1161/HYPERTENSIONAHA.110.153536. 20585108

[12] Larsen FJ, Ekblom B, Sahlin K, Lundberg JO, Weitzberg E. Effects of dietary nitrate on blood pressure in healthy volunteers. N Engl J Med 355: 2792–2793, 2006. doi:10.1056/NEJMc062800. 17192551

[13] Siervo M, Lara J, Ogbonmwan I, Mathers JC. Inorganic nitrate and beetroot juice supplementation reduces blood pressure in adults: a systematic review and meta-analysis. J Nutr 143: 818–826, 2013. doi:10.3945/jn.112.170233. 23596162

[14] Vanhatalo A, Bailey SJ, Blackwell JR, DiMenna FJ, Pavey TG, Wilkerson DP, Benjamin N, Winyard PG, Jones AM. Acute and chronic effects of dietary nitrate supplementation on blood pressure and the physiological responses to moderate-intensity and incremental exercise. Am J Physiol Regul Integr Comp Physiol 299: R1121–R1131, 2010. doi:10.1152/ajpregu.00206.2010. 20702806

[15] Webb AJ, Patel N, Loukogeorgakis S, Okorie M, Aboud Z, Misra S, Rashid R, Miall P, Deanfield J, Benjamin N, MacAllister R, Hobbs AJ, Ahluwalia A. Acute blood pressure lowering, vasoprotective, and antiplatelet properties of dietary nitrate via bioconversion to nitrite. Hypertension 51: 784–790, 2008. doi:10.1161/HYPERTENSIONAHA.107.103523. 18250365 PMC2839282

[16] Kapil V, Khambata RS, Jones DA, Rathod K, Primus C, Massimo G, Fukuto JM, Ahluwalia A. The noncanonical pathway for in vivo nitric oxide generation: the nitrate-nitrite-nitric oxide pathway. Pharmacol Rev 72: 692–766, 2020. doi:10.1124/pr.120.019240. 32576603

[17] Wagner DA, Young VR, Tannenbaum SR, Schultz DS, Deen WM. Mammalian nitrate biochemistry: metabolism and endogenous synthesis. IARC Sci Publ 57: 247–253, 1984. 6533015

[18] Qin L, Liu X, Sun Q, Fan Z, Xia D, Ding G, Ong HL, Adams D, Gahl WA, Zheng C, Qi S, Jin L, Zhang C, Gu L, He J, Deng D, Ambudkar IS, Wang S. Sialin (SLC17A5) functions as a nitrate transporter in the plasma membrane. Proc Natl Acad Sci USA 109: 13434–13439, 2012. doi:10.1073/pnas.1116633109. 22778404 PMC3421170

[19] Spiegelhalder B, Eisenbrand G, Preussmann R. Influence of dietary nitrate on nitrite content of human saliva: possible relevance to in vivo formation of N-nitroso compounds. Food Cosmet Toxicol 14: 545–548, 1976. doi:10.1016/s0015-6264(76)80005-3. 1017769

[20] Duncan C, Dougall H, Johnston P, Green S, Brogan R, Leifert C, Smith L, Golden M, Benjamin N. Chemical generation of nitric oxide in the mouth from the enterosalivary circulation of dietary nitrate. Nat Med 1: 546–551, 1995. doi:10.1038/nm0695-546. 7585121

[21] Govoni M, Jansson EÅ, Weitzberg E, Lundberg JO. The increase in plasma nitrite after a dietary nitrate load is markedly attenuated by an antibacterial mouthwash. Nitric Oxide 19: 333–337, 2008. doi:10.1016/j.niox.2008.08.003. 18793740

[22] Benjamin N, O'Driscoll F, Dougall H, Duncan C, Smith L, Golden M, McKenzie H. Stomach NO synthesis. Nature 368: 502, 1994. doi:10.1038/368502a0. 8139683

[23] Bailey SJ, Blackwell JR, Wylie LJ, Holland T, Winyard PG, Jones AM. Improvement in blood pressure after short-term inorganic nitrate supplementation is attenuated in cigarette smokers compared to non-smoking controls. Nitric Oxide 61: 29–37, 2016. doi:10.1016/j.niox.2016.10.002. 27744007

[24] Jansson EÅ, Huang L, Malkey R, Govoni M, Nihlén C, Olsson A, Stensdotter M, Petersson J, Holm L, Weitzberg E, Lundberg JO. A mammalian functional nitrate reductase that regulates nitrite and nitric oxide homeostasis. Nat Chem Biol 4: 411–417, 2008. doi:10.1038/nchembio.92. 18516050

[25] Lundberg JO. Nitrate transport in salivary glands with implications for NO homeostasis. Proc Natl Acad Sci USA 109: 13144–13145, 2012. doi:10.1073/pnas.1210412109. 22851765 PMC3421160

[26] Elishoov H, Wolff A, Kravel LS, Shiperman A, Gorsky M. Association between season and temperature and unstimulated parotid and submandibular/sublingual secretion rates. Arch Oral Biol 53: 75–78, 2008. doi:10.1016/j.archoralbio.2007.08.002. 17870051

[27] Ligtenberg AJM, Meuffels M, Veerman ECI. Effects of environmental temperature on saliva flow rate and secretion of protein, amylase and mucin 5B. Arch Oral Biol 109: 104593, 2020. doi:10.1016/j.archoralbio.2019.104593. 31710967

[28] Mylona E, Fahlman MM, Morgan AL, Boardley D, Tsivitse SK. s-IgA response in females following a single bout of moderate intensity exercise in cold and thermoneutral environments. Int J Sports Med 23: 453–456, 2002. doi:10.1055/s-2002-33744. 12215966

[29] Burleigh M, Liddle L, Muggeridge DJ, Monaghan C, Sculthorpe N, Butcher J, Henriquez F, Easton C. Dietary nitrate supplementation alters the oral microbiome but does not improve the vascular responses to an acute nitrate dose. Nitric Oxide 89: 54–63, 2019. doi:10.1016/j.niox.2019.04.010. 31051259

[30] Cocksedge SP, Causer AJ, Winyard PG, Jones AM, Bailey SJ. Oral temperature and pH influence dietary nitrate metabolism in healthy adults. Nutrients 15: 784, 2023. doi:10.3390/nu15030784. 36771490 PMC9919366

[31] Wylie LJ, Kelly J, Bailey SJ, Blackwell JR, Skiba PF, Winyard PG, Jeukendrup AE, Vanhatalo A, Jones AM. Beetroot juice and exercise: pharmacodynamic and dose-response relationships. J Appl Physiol (1985) 115: 325–336, 2013. doi:10.1152/japplphysiol.00372.2013. 23640589

[32] Notay K, Incognito AV, Millar PJ. Acute beetroot juice supplementation on sympathetic nerve activity: a randomized, double-blind, placebo-controlled proof-of-concept study. Am J Physiol Heart Circ Physiol 313: H59–H65, 2017. doi:10.1152/ajpheart.00163.2017. 28476923

[33] Cosby K, Partovi KS, Crawford JH, Patel RP, Reiter CD, Martyr S, Yang BK, Waclawiw MA, Zalos G, Xu X, Huang KT, Shields H, Kim-Shapiro DB, Schechter AN, Cannon RO 3rd, Gladwin MT. Nitrite reduction to nitric oxide by deoxyhemoglobin vasodilates the human circulation. Nat Med 9: 1498–1505, 2003. doi:10.1038/nm954. 14595407

[34] Bojic D, Bojic A, Perovic J. The effects of dietary nitrate, pH and temperature on nitrate reduction in the human oral cavity. Phys, Chem Technol 3: 53–60, 2004. doi:10.2298/FUPCT0401053B.

[35] Arnold JT, Bailey SJ, Hodder SG, Fujii N, Lloyd AB. Independent and combined impact of hypoxia and acute inorganic nitrate ingestion on thermoregulatory responses to the cold. Eur J Appl Physiol 121: 1207–1218, 2021. doi:10.1007/s00421-021-04602-x. 33558988 PMC7966143

[36] Eglin CM, Costello JT, Bailey SJ, Gilchrist M, Massey H, Shepherd AI. Effects of dietary nitrate supplementation on the response to extremity cooling and endothelial function in individuals with cold sensitivity. A double blind, placebo controlled, crossover, randomised control trial. Nitric Oxide 70: 76–85, 2017. doi:10.1016/j.niox.2017.09.005. 28941934

[37] Wakabayashi H, Sugiyama K, Suzuki S, Sakihama Y, Hashimoto M, Barwood MJ. Influence of acute beetroot juice supplementation on cold-induced vasodilation and fingertip rewarming. Eur J Appl Physiol 123: 495–507, 2023. doi:10.1007/s00421-022-05071-6. 36305974

[38] Wickham KA, Steele SW, Cheung SS. Effects of acute dietary nitrate supplementation on cold-induced vasodilation in healthy males. Eur J Appl Physiol 121: 1431–1439, 2021. doi:10.1007/s00421-021-04621-8. 33620545

[39] Ship JA, Fischer DJ. The relationship between dehydration and parotid salivary gland function in young and older healthy adults. J Gerontol Ser A Biol Sci Med Sci 5: M310–M319, 1997. doi:10.1093/gerona/52A.5.M310. 9310086

[40] Minshull C, James L. The effects of hypohydration and fatigue on neuromuscular activation performance. Appl Physiol Nutr Metab 38: 21–26, 2013. doi:10.1139/apnm-2012-0189. 23368824

[41] Lee A, Guest S, Essick G. Thermally evoked parotid salivation. Physiol Behav 87: 757–764, 2006. doi:10.1016/j.physbeh.2006.01.021. 16529781

[42] Granli T, Dahl R, Brodin P, Bøckman OC. Nitrate and nitrite concentrations in human saliva: variations with salivary flow-rate. Food Chem Toxicol 27: 675–680, 1989. doi:10.1016/0278-6915(89)90122-1. 2606404

[43] Lee JK, Maughan RJ, Shirreffs SM. The influence of serial feeding of drinks at different temperatures on thermoregulatory responses during cycling. J Sports Sci 26: 583–590, 2008. doi:10.1080/02640410701697388. 18344129

[44] Winslow C-EA, Herrington LP, Gagge AP. A new method of partitional calorimetry. Am J Physiol 3: 495–69, 1936. doi:10.1152/ajplegacy.1936.116.3.641.

[45] Mitchell D, Wyndham CH. Comparison of weighting formulas for calculating mean skin temperature. J Appl Physiol 26: 616–622, 1969. doi:10.1152/jappl.1969.26.5.616. 5781615

[46] Liddle L, Monaghan C, Burleigh MC, McIlvenna LC, Muggeridge DJ, Easton C. Changes in body posture alter plasma nitrite but not nitrate concentration in humans. Nitric Oxide. 72: 59–65, 2018. doi:10.1016/j.niox.2017.11.008. 29199111

[47] Kariyawasam AP, Dawes C. A circannual rhythm in unstimulated salivary flow rate when the ambient temperature varies by only about 2 degrees C. Arch Oral Biol 50: 919–922, 2005. doi:10.1016/j.archoralbio.2005.03.001. 16137501

[48] Kavanagh DA, O'Mullane DM, Smeeton N. Variation of salivary flow rate in adolescents. Arch Oral Biol 43: 347–352, 1998. doi:10.1016/s0003-9969(98)00020-x. 9681109

[49] Louridis O, Demetriou N, Bazopoulou-Kyrkanides E. Environmental temperature effect on the secretion rate of “resting” and stimulated human mixed saliva. J Dent Res 49: 1136–1140, 1970. doi:10.1177/00220345700490052301. 5272096

[50] Bahadoran Z, Mirmiran P, Carlström M, Ghasemi A. Inorganic nitrate: a potential prebiotic for oral microbiota dysbiosis associated with type 2 diabetes. Nitric Oxide 116: 38–46, 2021. doi:10.1016/j.niox.2021.09.001. 34506950

[51] Proctor GB, Carpenter GH. Regulation of salivary gland function by autonomic nerves. Auton Neurosci 133: 3–18, 2007. doi:10.1016/j.autneu.2006.10.006. 17157080

[52] Burleigh MC, Sculthorpe N, Henriquez FL, Easton C. Nitrate-rich beetroot juice offsets salivary acidity following carbohydrate ingestion before and after endurance exercise in healthy male runners. PLoS One 15: e0243755, 2020. doi:10.1371/journal.pone.0243755. 33320868 PMC7737958

[53] Burleigh MC, Liddle L, Monaghan C, Muggeridge DJ, Sculthorpe N, Butcher JP, Henriquez FL, Allen JD, Easton C. Salivary nitrite production is elevated in individuals with a higher abundance of oral nitrate-reducing bacteria. Free Radic Biol Med 120: 80–88, 2018. doi:10.1016/j.freeradbiomed.2018.03.023. 29550328

[54] McDonagh STJ, Wylie LJ, Webster JMA, Vanhatalo A, Jones AM. Influence of dietary nitrate food forms on nitrate metabolism and blood pressure in healthy normotensive adults. Nitric Oxide 72: 66–74, 2018. doi:10.1016/j.niox.2017.12.001. 29223585

[55] Woessner M, Smoliga JM, Tarzia B, Stabler T, Van Bruggen M, Allen JD. A stepwise reduction in plasma and salivary nitrite with increasing strengths of mouthwash following a dietary nitrate load. Nitric Oxide 54: 1–7, 2016. doi:10.1016/j.niox.2016.01.002. 26778277

[56] Bailey SJ, Blackwell JR, Wylie LJ, Emery A, Taylor E, Winyard PG, Jones AM. Influence of iodide ingestion on nitrate metabolism and blood pressure following short-term dietary nitrate supplementation in healthy normotensive adults. Nitric Oxide 63: 13–20, 2017. doi:10.1016/j.niox.2016.12.008. 28024935

[57] Jin L, Zhang M, Xu J, Xia D, Zhang C, Wang J, Wang S. Music stimuli lead to increased levels of nitrite in unstimulated mixed saliva. Sci China Life Sci 61: 1099–1106, 2018. doi:10.1007/s11427-018-9309-3. 29934916

[58] Jackson JK, Patterson AJ, MacDonald-Wicks LK, Oldmeadow C, McEvoy MA. The role of inorganic nitrate and nitrite in cardiovascular disease risk factors: a systematic review and meta-analysis of human evidence. Nutr Rev 76: 348–371, 2018. doi:10.1093/nutrit/nuy005. 29506204

[59] Korhonen I. Blood pressure and heart rate responses in men exposed to arm and leg cold pressor tests and whole-body cold exposure. Int J Circumpolar Health 65: 178–184, 2006. doi:10.3402/ijch.v65i2.18090. 16711469

[60] Komulainen S, Tähtinen T, Rintamäki H, Virokannas H, Keinänen-Kiukaanniemi S. Blood pressure responses to whole-body cold exposure: effect of carvedilol. Eur J Clin Pharmacol 56: 637–642, 2000. doi:10.1007/s002280000208. 11214769

[61] de Vries CJ, DeLorey DS. Effect of acute dietary nitrate supplementation on sympathetic vasoconstriction at rest and during exercise. J Appl Physiol (1985). 127: 81–88, 2019. doi:10.1152/japplphysiol.01053.2018. 31095461 PMC6692739

[62] Feelisch M, Akaike T, Griffiths K, Ida T, Prysyazhna O, Goodwin JJ, Gollop ND, Fernandez BO, Minnion M, Cortese-Krott MM, Borgognone A, Hayes RM, Eaton P, Frenneaux MP, Madhani M. Long-lasting blood pressure lowering effects of nitrite are NO-independent and mediated by hydrogen peroxide, persulfides, and oxidation of protein kinase G1α redox signalling. Cardiovasc Res 116: 51–62, 2020. doi:10.1093/cvr/cvz202. 31372656 PMC6918062

[63] Pinheiro LC, Amaral JH, Ferreira GC, Portella RL, Ceron CS, Montenegro MF, Toledo JC Jr, Tanus-Santos JE. Gastric S-nitrosothiol formation drives the antihypertensive effects of oral sodium nitrite and nitrate in a rat model of renovascular hypertension. Free Radic Biol Med 87: 252–262, 2015. doi:10.1016/j.freeradbiomed.2015.06.038. 26159506

[64] Wei C, Vanhatalo A, Kadach S, Stoyanov Z, Abu-Alghayth M, Black MI, Smallwood MJ, Rajaram R, Winyard PG, Jones AM. Reduction in blood pressure following acute dietary nitrate ingestion is correlated with increased red blood cell S-nitrosothiol concentrations. Nitric Oxide 138-139: 1–9, 2023. doi:10.1016/j.niox.2023.05.008. 37268184

[65] Oliveira-Paula GH, Tanus-Santos JE. Nitrite-stimulated gastric formation of S-nitrosothiols as an antihypertensive therapeutic strategy. Curr Drug Targets 20: 431–443, 2019. doi:10.2174/1389450119666180816120816. 30112990

[66] Shepherd AI, Costello JT, Bailey SJ, Bishop N, Wadley AJ, Young-Min S, Gilchrist M, Mayes H, White D, Gorczynski P, Saynor ZL, Massey H, Eglin CM. “Beet” the cold: beetroot juice supplementation improves peripheral blood flow, endothelial function, and anti-inflammatory status in individuals with Raynaud’s phenomenon. J Appl Physiol (1985) 127: 1478–1490, 2019. doi:10.1152/japplphysiol.00292.2019. 31343948 PMC6879832

[67] Arnold JT, Lloyd AB, Bailey SJ, Fujimoto T, Matsutake R, Takayanagi M, Nishiyasu T, Fujii N. The nitric oxide dependence of cutaneous microvascular function to independent and combined hypoxic cold exposure. J Appl Physiol (1985) 129: 947–956, 2020. doi:10.1152/japplphysiol.00487.2020. 32881624

[68] Alpérovitch A, Lacombe JM, Hanon O, Dartigues JF, Ritchie K, Ducimetière P, Tzourio C. Relationship between blood pressure and outdoor temperature in a large sample of elderly individuals: the three-city study. Arch Intern Med 169: 75–80, 2009. doi:10.1001/archinternmed.2008.512. 19139327

[69] Minami J, Kawano Y, Ishimitsu T, Yoshimi H, Takishita S. Seasonal variations in office, home and 24 h ambulatory blood pressure in patients with essential hypertension. J Hypertens 14: 1421–1425, 1996. doi:10.1097/00004872-199612000-00006. 8986924

[70] Lloyd EL. The role of cold in ischaemic heart disease: a review. Public Health 105: 205–215, 1991. doi:10.1016/S0033-3506(05)80110-6. 2062993

[71] Sheth T, Nair C, Muller J, Yusuf S. Increased winter mortality from acute myocardial infarction and stroke: the effect of age. J Am Coll Cardiol 33: 1916–1919, 1999. doi:10.1016/S0735-1097(99)00137-0. 10362193

[72] Liddle L, Monaghan C, Burleigh MC, Baczynska KA, Muggeridge DJ, Easton C. Reduced nitric oxide synthesis in winter: a potential contributing factor to increased cardiovascular risk. Nitric Oxide 127: 1–9, 2022. doi:10.1016/j.niox.2022.06.007. 35792235

